# Editorial

**DOI:** 10.1120/jacmp.v7i4.2444

**Published:** 2006-11-28

We have some changes this quarter. It gives me great pleasure to welcome new Associate Editors: John Antolak, Ivan Brezovich, and Benjamin Nelms. The JACMP submission rate continues to grow, especially in Radiation Oncology Physics, and we are most fortunate to have these extraordinary individuals working with us. Welcome to each of you. In addition, we welcome a new Manuscript Manager, Bonnie Ratcliff, and a new Copy Editor, Ann Fothergill‐Brown. The JACMP simply will not function unless we have talented and diligent individuals in these positions, so thanks to both of you for all you do for the JACMP. Also, I wish to announce that Jim Smathers will be retiring as Book Review Editor for the JACMP. Jim has served the JACMP in this capacity since its inception, and I want to express my thanks for his extraordinary service for the past seven years.

This issue, I would like to announce the JACMP is now co‐sponsored and is an official journal of the International Organization of Medical Physics. This is an important milestone as we seek to expand the international reach of the JACMP. We are pleased that the JACMP is available without cost to any physicist worldwide who has web access. We hope to continue our mission to benefit the patient with such clinical information.

I am very pleased to announce that the JACMP is now a Thomson‐ISI journal and will be abstracted in Current Contents, beginning with the Volume 6 Number 1 issue (Winter 2005). At some point we expect to receive an impact factor and when this happens, we will make it available. Let me offer many thanks to all our Associate Editors and Reviewers for all efforts to make the JACMP a journal worthy of this honor.

This quarter, it is my pleasure to welcome a guest editorial from Peter R. Almond, Ph.D., whom I consider a special mentor and friend. Many of you will remember that Peter was the first Editor‐in‐Chief of the JACMP. He has a special interest in the history of medical physics, and I hope you enjoy his story below.

Michael D. Mills, PhD

Editor‐in‐Chief

## Grimmett and the Cobalt Unit

Peter R. Almond, Ph.D.

Leonard George Grimmett (1903 to 1951) was an English medical physicist who, in 1948, was recruited by The University of Texas M. D. Anderson Hospital for Cancer Research in Houston, Texas to organize the new institution's physics department. He arrived in Houston in early 1949 and quickly became involved in efforts to design and build a cobalt‐60 (Co‐60) treatment unit. In October 1950, he became the first person to publish the design of a cobalt machine;^(1)^ however, he had begun thinking about such a machine many years prior.

January 2007 will mark the seventieth anniversary of the publication of a long‐forgotten, rediscovered article that might be considered the concept paper for the Co‐60 unit. The paper was titled, “Radium Beam Therapy and High‐Voltage X‐rays.” It was authored by A.S. Eve and L. G. Grimmett from the Radium Beam Therapy Research in London, and it appeared in the January 9, 1937 issue of *Nature*.^(2)^

The group, with the awkward sounding name (“Radium Beam Therapy Research”) had been established in London in 1934 to investigate the use of beam therapy using gamma rays from radium in the treatment of cancer. The idea was not new; however, at that time, the amount of radium being used was about 4 grams. It took 30 minutes or longer to deliver each treatment with this amount of radium. Thirty minutes was the longest period of time patients could be expected to lie still, thus it was not unusual for patients to undergo two treatment sessions per day to deliver the desired dose to the tumor.

Radium Beam Therapy Research was the brainchild of Professor Cunningham McLennan. He had been chairman of the physics department at Toronto University but had retired to England in 1932. In Canada he had served on the Canadian Radium Commission and in London he became a member of a committee that was researching and reporting on the value of beam therapy using radium. McLennan was convinced that larger amounts of radium were needed in order to conduct a valid study, and he proposed the formation of the Radium Beam Therapy Research unit. It would be located at the Radium Institute in London as a cooperative effort with the Royal Cancer Hospital. McLennan negotiated with the Union Miniére du Haut Katanga of Belgium for the loan of the radium, initially 5 grams and eventually 10 grams. This was not an insignificant contribution, with radium costing $\underset{˙}{\overset{\sim }{£}}1$ per milligram at that time ($1000 in 2006 U.S. currency).

Grimmett was chosen to be the physicist for the Radium Beam Therapy Research unit. He had begun his career as a medical physicist in 1929 at the Westminster Hospital in London, where he was involved with the design and use of two radium units using 4 grams of radium. The Radium Commission, however, owned the radium, and in 1932, they divided the 4 grams into four 1‐gram sources. Westminster Hospital retained 1 gram, and the rest went to other hospitals. This essentially made beam therapy with radium impossible. Grimmett was pleased to join a group that would have a sufficient amount of radium with which to design a practical radium unit.

Grimmett visited Rolf Sievert, a medical physicist in Stockholm in 1933 to see the radium treatment machine that he had just designed^(3)^. McLennan also visited Sievert, and he and Grimmett decided that the Radium Beam Therapy Research would use the same approach in the design of their radium treatment machine as had been used by the Stockholm group. Thus, the Grimmett's machine is very similar to Sievert's^(4)^; however, Grimmett introduced the unique concept of pneumatically moving the radium source from the storage safe to the treatment head. The Grimmett unit was initially loaded with 5 grams of radium and then with 10 grams.

To oversee the work of the Radium Beam Therapy Research a governing board of distinguished scientists was established, and Ernest Rutherford served as the first Honorary Physicist. Rutherford had won the 1908 Noble Prize in chemistry for his investigations into the disintegration of radioactive substances, including radium. Arthur Eve stepped into the position when Rutherford retired. Eve had been chairman of the physics department at McGill University, Montreal, Quebec, from 1919 to 1935 and had worked there with Rutherford. Eve had also retired to London. He was not involved with the day‐to‐day work of the Radium Beam Therapy Research group, but as the senior physicist on the executive committee, he was included as an author of the January 1937 *Nature* article, with his name appearing first as a courtesy. His name also ensured speedy acceptance of the paper by *Nature*. However, the ideas and work reported in the paper were all Grimmett's. The paper assessed the biologic advantage to megavoltage gamma‐rays compared to the X‐rays produced by a high‐voltage X‐ray tube. In the paper, Grimmett wrote:

*“Many radiologists believe that gamma‐ray therapy is superior to X‐ray therapy in its biological effects, and they attribute this superiority to the shorter wave‐length of the gamma‐rays; encouraged by this belief, they are striving after X‐rays generated at higher and higher voltages, which approach the gamma‐rays of radium in their nature.”* ^(2)^

He went on to show that even with a tube operating at 1‐million volts, there would be no X‐rays of that energy, whereas radium has quite a few gamma rays in the 1‐ to 2‐MeV range. To match these gamma rays with comparable X‐ray energies, Grimmett wrote, would require a 3‐million‐volt tube, and he was not sure that that was possible. He was also concerned that the cost of the radium limited its use in beam therapy.

What he did realize was that the output of the X‐ray tubes was much higher than could be obtained with radium, and that therefore, treatments with X‐rays could be given at extended source‐to‐surface distances (SSD) that gave a superior depth dose compared to that of gamma rays. Gimmett gave the following example: For a 370‐kv tube at a treatment distance of 75‐cm, the depth dose at 10 cm was 43%. For a radium source at 5‐cm SSD, the depth dose at 10 cm was 11%. He concluded that:

*“The fact is that both radium and X‐ray treatments are governed by the inverse square law, and that the superior penetrating power of gamma‐rays cannot be exploited unless prohibitive quantities of radium are available to make it possible to work with large radium‐skin distances…*

***It is possible that in a few years time the new discoveries of physics…artificial radioactivity, will find a place in radiation therapy… it is now possible to obtain gamma‐rays from artificial radioactive substances with energies far in excess of anything radium emits…if it is possible to make it cheaply in bulk, it could be inserted…into a radium unit of conventional design and used for treatment in place of radium.“*** ^(2)^ (Emphasis present author)

In the *Nature* paper, Grimmett gave radio‐sodium as an example of an artificial radioactive isotope. Although radio‐sodium has a very short half‐life, he suggested that the source might be exchanged daily to maintain a sufficiently high output. Not enough was known about Co‐60 at that time for Grimmett to suggest it, but the idea clearly stayed with him. In his memoirs, Marshal Bruce, who later collaborated with Grimmet, recalled that the idea that Co‐60 might be a suitable replacement for radium first occurred to Grimmett while he was reading *Physical Review* in an air‐raid shelter during World War II.^(5)^ It is known that Grimmett's house in the suburbs of London was damaged by a flying bomb and that he took refuge in his own home‐built air‐raid shelter during such attacks. This would have been between mid‐1944 and early 1945. During the 1930s several investigators published reports and papers concerning cobalt‐induced radioactivity. The first was by Rotblat in *Nature* in 1935^(6)^. Initially there was much confusion about the emitted radiations, especially the half‐life and energies of the gamma‐rays, probably because of the impurities in the cobalt and a competing 10‐minute half‐life isomeric transition.

Sampson, Ridenour, and Bleakney,^(7)^ in 1936, first observed a long‐lived isotope of Co‐60 by irradiating Co‐59 with neutrons. They reported the half‐life of this isotope to be more than 1 year. Three other papers, however, are likely among the ones Grimmett read in his air‐raid shelter. One of these articles was perhaps an article by Risser^(8)^ entitled “Neutron‐Induced Radioactivity of Long Life in Cobalt” published in October 1937. He originally reported that there was only 1 gamma‐ray with a value of 1.5 to 2.0 MeV. The actual values of two gamma rays are 1.17 and 1.33 MeV. Risser did not have enough activity to measure the half‐life accurately, so he approximated it to be 2.00±0.5 years (actual value 5.26 years). In another article, Livingood and Seaborg^(9)^ reported producing radioactive cobalt in the cyclotron using deuteron bombardment and also using a radium beryllium neutron source to irradiate Co‐59. They also measured the energy of the high‐energy gamma rays as 1.3 MeV; however, their estimate of the half‐life was in accurate, believing a value of over 10 years was indicated. The third article from *Physical Review* that might have impressed Grimmett was a report by Nelson and colleagues^(10)^ that identified the half‐life of Co‐60 as 5.3 years.

Here then was evidence of a radioactive isotope with the right energy gamma rays and with a half‐life that would allow the treatment machine to be used for several years without replacing the radioactive source. Although the published values of the energies of the gamma rays (1.3 to 2.0 MeV) were inaccurate, they were sufficiently high enough for Grimmett to realize the potential efficacy of Co‐60. No one, not even Grimmett, knew then how, how much, and at what cost Co‐60 could be produced.

After the war, Grimmett most likely read the paper by J. S. Mitchell^(11)^ published in the December 1946 issue of the *British Journal of Radiology*. This paper is often cited as having initiated the Co‐60 era. Mitchell specifically suggested Co‐60 as a replacement for radium beam therapy, and he reported its half‐life as 5.3 years and its gamma ray energies as 1.3 MeV and 1.1 MeV. He also reported that it could be produced in “the pile” (nuclear reactor). But it was still unclear at that time whether the isotope could be produced in the quantities required and what quantities were actually needed. If radium was the guide, at least 10 to 50 curies per treatment unit might be suitable. The following excerpt from Brucer's memoirs concerning the year 1949^(5)^:

*“…Grimmett was radiation physicist at Houston's cancer hospital, not yet a citizen. I had just been appointed chairman of the Oak Ridge isotope research hospital and was looking for ideas. I stopped off to see Grimmett on my way to the super‐secret city of Oak Ridge and was given a complete history of all the warts on the radium bomb. Co‐60 might be, Grimmett said, the answer to cancer. I invited him to Oak Ridge.”*

Brucer was the newly appointed head of the medical division of the Oak Ridge Institute for Nuclear Studies (ORINS). At the time, he was commuting between Galveston, TX (where he had previously held an appointment at The University of Texas School of Medicine) and Oak Ridge, TN. In late 1949, Brucer arranged for Grimmett to visit Oak Ridge secretly. Grimmett was not a U.S. citizen, and the ORINS was under tight security, so special precautions had to be made. Brucer and Gimmett met with Paul Aebersold, then head of the Isotope Division of the U.S. Atomic Energy Commission, in an area of the research hospital that was still under construction. Brucer said that Grimmett initially asked for 10 curies of Co‐60^(5)^, probably because it would have been analogous to the 10 grams of radium that was then being used in the radium irradiators that Grimmett had designed before the war. Abersold thought that a few hundred curies might be available, and Brucer rounded it out to an even 1,000 curies. When Grimmett realized that 1,000 curies was possible, he went back to Houston and designed a unit based on that amount, taking full advantage of the benefits to be derived from such a large amount of activity, as described below.

By the end of 1949, the medical division of ORINS and M. D. Anderson Hospital for Cancer Research had prepared a joint proposal to the Atomic Energy Commission for the design and construction of a 1,000‐curie Co‐60 therapy unit. Grimmett described this unit in a paper published in 1950^(1)^. The paper was a reprieve of his 1937 paper: both compared the radiation spectra of a high‐voltage X‐ray tube and radium, but in the newer paper, the gamma rays of Co‐60 were included in a figure showing the X‐ray spectrum and the radium and Co‐60 gamma‐ray lines. Grimmett knew well the characteristics of Co‐60. The values he quoted for the gamma ray energies, half‐life, and exposure rate constant were similar to the accepted values used today. The unit, however, was a far cry from the simple suggestion he made in his 1937 paper that the artificial radioactivity*, “… could be inserted…into a radium unit of conventional design and used for treatment in place of radium.”* ^(2)^

With 1,000 curies, the source could be moved further away from the patient surface than the 5 to 10 cm required with the radium units; Grimmett chose a 50‐cm distance. This unit would therefore have a superior depth‐dose compared with the kilo‐voltage X‐ray machines then in use, fulfilling one of the advantages he had suggested in the 1937 paper. In 1950, he wrote, *“…Cobalt‐60 may be considered ‘equivalent’ to a 2 MeV X‐ray tube.”* ^(1)^ He also designed the unit with a small source size, a 2‐cm cube, arguing that with the extended treatment distance and a smaller source size, the radiation beam produced by the unit would have a much smaller, better‐defined penumbra—a feature absent in the radium units. He understood that it would be inherently dangerous to move 1,000 curies from storage to the treatment unit pneumatically, as he had done with his radium units. *“The pneumatic system of propelling the radioactive material by air pressure to and from a storage safe was considered and rejected because it may on rare occasions break down. A breakdown with 1000 curies of Cobalt‐60 would be intolerable.”* ^(1)^

The new unit, therefore, was self contained, with sufficient shielding to make it safe to work around while setting up patients with the leakage radiation not exceeding the then permissible dose rate of 0.3 roentgens per week.

The concluding paragraph of Grimmett's 1959 paper is of interest.

*“It is our eventual hope to produce a simple, cheap, and reliable machine, needing no servicing or replacement, apart from the replenishing of the source every five years or so, which will enable monochromatic gamma‐rays to be tried for the first time in cancer treatment. The cost is difficult to estimate at this stage, but will probably be in the region of $30,000. It would seem to be a sound way of using atomic products, which should bring the benefits of high‐voltage radiation within the reach of the ordinary hospital.”* ^(1)^

Although overly optimistic, especially with regard to the cost, Grimmett's predictions about the use of Co‐60 units proved true. Thousands of units have been built and used worldwide, and hundreds are still in use. Millions of patients have been treated on them. Grimmett never saw his unit in use. He died suddenly May 1951 as it was being completed; however, it was used clinically at M. D. Anderson until 1963. The unit was eventually loaded with a 2,000‐curie source, and the treatment distance was extended to 75 cm.

Other people played important roles in the development of Co‐60 units, notably Dr. Harold Johns in Canada. Johns and Grimmett met in early 1950 at a meeting in Washington D.C., which Brucer had arranged to discuss cobaltCo‐60 treatment machines. Clearly, however, the idea of replacing radium with a suitable artificial radioactive material was in Grimmett's mind long before he arrived in Houston.

Peter R Almond

The University of Texas M.D. Anderson Cancer Center Houston, Texas

**Table:** 

**The Journal of Applied Clinical Medical Physics**	
**Editorial Board 2006**	
Editor‐in‐Chief	Michael Mills
Deputy Editor‐in‐Chief	Timothy Solberg
**Associate Editors**	
Radiation Oncology Physics	Nzhde Agazaryan, Salahuddin Ahmad
	John Antolak, Ivan Brezovich
	Jefferson Fairbanks, B. Gino Fallone
	Janelle Molloy, Benjamin Nelms
	Matthew Podgorsak, Nikos Papanikolaou
	William Salter, Mehrdad Sarfaraz
	Lu Wang, Albert Zacarias
Medical Imaging	Geoffrey Clarke, William Pavlicek
Radiation Measurements	Larry DeWerd
Radiation Protection & Regulations	James Rodgers
Nonionizing Topics	Bhudatt Paliwal
Other Topics	Timothy Solberg
Book/Media Reviews	James Smathers
Editorials	John Horton
**Journal Production Team**	
Proofreader	Heather Shand
Copy Editor	Ann Fothergill‐Brown
Layout Editor	Laura Shand
Manuscript Manager	Bonnie Ratcliff
